# Classification of Acoustic Influences Registered with Phase-Sensitive OTDR Using Pattern Recognition Methods

**DOI:** 10.3390/s23020582

**Published:** 2023-01-04

**Authors:** Ivan A. Barantsov, Alexey B. Pnev, Kirill I. Koshelev, Vadim S. Tynchenko, Vladimir A. Nelyub, Aleksey S. Borodulin

**Affiliations:** 1 Photonics and Infra-Red Technology Scientific Education Center, Bauman Moscow State Technical University, 105005 Moscow, Russia; 2 Artificial Intelligence Technology Scientific and Education Center, Bauman Moscow State Technical University, 105005 Moscow, Russia

**Keywords:** acoustics, OTDR, classification, pattern recognition, convolutional neural network, AlexNet, DenseNet169, ResNet50

## Abstract

This article is devoted to the development of a classification method based on an artificial neural network architecture to solve the problem of recognizing the sources of acoustic influences recorded by a phase-sensitive OTDR. At the initial stage of signal processing, we propose the use of a band-pass filter to collect data sets with an increased signal-to-noise ratio. When solving the classification problem, we study three widely used convolutional neural network architectures: AlexNet, ResNet50, and DenseNet169. As a result of computational experiments, it is shown that the AlexNet and DenseNet169 architectures can obtain accuracies above 90%. In addition, we propose a novel CNN architecture based on AlexNet, which obtains the best results; in particular, its accuracy is above 98%. The advantages of the proposed model include low power consumption (400 mW) and high speed (0.032 s per net evaluation). In further studies, in order to increase the accuracy, reliability, and data invariance, the use of new algorithms for the filtering and extraction of acoustic signals recorded by a phase-sensitive reflectometer will be considered.

## 1. Introduction

In this work, we consider a method for processing and classifying mechanical impacts, based on the analysis of acoustic waves recorded with a fiber optical sensor system using a phase-sensitive optical time-domain reflectometer (OTDR) that is described in the paper by Pnev, A.B. et al. [1]. Such sensor systems can be used to check the maintenance status of gas and oil pipelines (Svelto, C., Pniov, A. et al. [2]), guaranteeing a high probability of detecting suspicious leaks. Moreover, these systems can be used to monitor trains, as noted in the paper by Kovarik S. et al. [3]. With the help of fiber optic acoustic monitoring systems, it is possible to monitor runways (Merlo, S. et al. [4]) and perform the monitoring of vibrations of the optical fiber of the submarine cable for its safety (Fouda, B. [5]). Furthermore, these systems may be used for perimeter security.

The advantages of fiber optic sensor systems include

- The possibility of detecting many influences in several places at once, with a small error in determining the coordinates;
- Obtaining high information content about the source of acoustic influences.

In view of the above benefits, these systems require high-quality input signal processing.

Acoustic signals generated by mechanical impacts reflect the characteristics of their sources. The difficulty in classifying such signals is determining the best ways to extract features, as acoustic signals are typically non-stationary and manual feature extraction requires expert knowledge. The largest signal-to-noise ratio of various mechanical impacts occurs at certain frequencies; therefore, using a band-pass filter to extract vibration signals and automatically extracting features from the filtered data using a convolution neural network (CNN) [6,7,8,9] may provide an effective method for the extraction of features.

This work is carried out to create more advanced distributed acoustic sensor-based (DAS-based) perimeter detection systems that can detect acoustic signals generated by human steps. Cases are considered when a person walks and sneaks along and across the DAS.

Similar work has been carried out by Xu, C. et al. [10], where the short-time Fourier transform typically used for acoustic spectrum analysis was used for feature extraction, in order to obtain a time–frequency diagram. Next, a background subtraction spectrum was applied to remove broadband noise. A data set was then created, based on the obtained data. To automatically extract the necessary features, a CNN with an AlexNet-based architecture was used. The classification algorithm includes three-channel stacking:

- A soft-max layer takes the output of a fully connected layer;
- A support vector machine (SVM) algorithm takes the output from a fully connected layer;
- The SVM algorithm takes input data that are fed to the input of a fully connected layer.

In [11], Sun, Q. et al. carried out data feature extraction using pixel intensity histograms that have different distributions with respect to each mechanical impact. The SVM algorithm was used as a classifier.

Since the processed signals are a temporary process, solutions have been proposed using various types of RNN [12,13].

In [14,15,16,17], other algorithms for automatic feature extraction using ML models are described.

In [18,19,20,21], similar studies have been carried out to improve the quality of the signal, extract features, and develop algorithms to classify acoustic effects recorded by a phase-sensitive reflectometer. In the considered works, studies on simplified data augmentation, taking into account the preservation of the physical meaning of the signals, were not carried out. Essentially, in the works, the classification was carried out only between classes, among which background acoustic effects and signals distorted by interference were not taken into account. The existing solutions did not consider the issue of the computational complexity of the developed algorithm.

However, CNNs have some drawbacks, such as their training requiring a large amount of weakly correlated data. In this paper, as one of the solutions to increase the size of the data set, we consider the generation of additional data by making changes to existing data, which also makes it possible to balance classes, thus increasing the correctness of the classifier and the reliability of the assessment of the prediction quality. Furthermore, the CNN must be resistant to various external influences that complicate the classification of the desired acoustic waves. In addition, optical fiber sensor systems process data in real time using low-power hardware, thus limiting the algorithm’s computational complexity. Using a longer distributed sensor length allows for the monitoring of longer areas, increasing the economic efficiency of the system. Among the negative factors associated with increasing the length of the sensor is an increase in the amount of information processed over a period of time, which also contributes to limitations on the computational complexity of the algorithm.

The purpose of this work is to create a block to process and categorize the ADC signals of such a sensor system. The unit must perform classification with great accuracy, have low computational complexity, and operate smoothly on low-power computers.

## 2. Materials and Methods

### 2.1. Input Data

**Figure 1:**
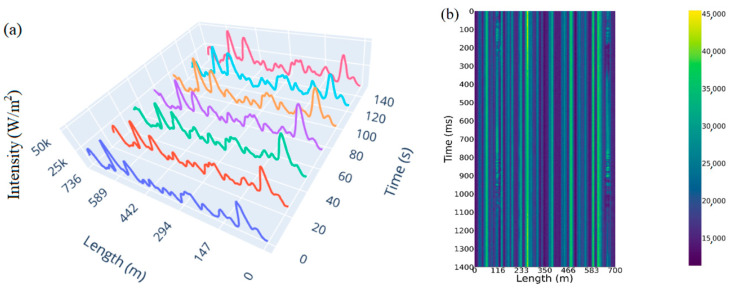
(**a**) Three-dimensional representation of a set of signal traces; and (**b**) an image of the space–time diagram, using a color map.

In a phase-sensitive reflectometer, the coherence length of the source is longer than the pulse duration, due to which the radiation scattered from inhomogeneities within the pulse duration is added, taking the phases into account. These phases for each wave can be considered as random variables, as a result of which the recorded scatter signal (Figure 1a) shows fluctuations. This signal is a one-dimensional analogue of the speckle patter.

These received signal traces’ deviations remain approximately until the phases of the scattering centered on any section of the cable are changed. This occurs when the optical fiber is deformed, which can be caused either by a direct impact on the cable, or by an acoustic wave that has reached it from environmental events. Thus, by analyzing the stability of the signal traces, it is possible to draw conclusions regarding the events that have occurred around the sensor.

From received signal traces with a sampling rate of 1000 Hz, we obtain a space–time matrix (Figure 1b), which can be considered as a single-channel image. In this project, the data are updated from the top row of the matrix to the bottom along the time axis. This type of data representation is called a waterfall.

An example of a classifiable action is a human step (approaching/retreating and walking along the fiber sensor).

### 2.2. Filtering

**Figure 2:**
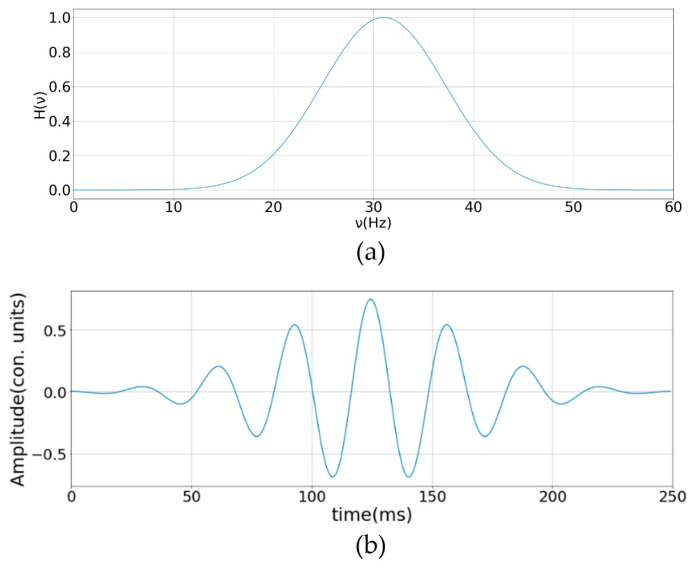
Frequency function of the designed filter (frequency axis is scaled) (**a**) and created kernel function (**b**) used in impulse response sense.

A band-pass filter was used to extract the data. The frequency function of the filter (Figure 2) was generated in such a way as to highlight the temporal frequencies at which the ratio of the recorded acoustic signals of steps to the background signal would be maximal for various events. The impulse response of the filter was generated using the Morlet wavelet, which Russell, B. et al. describe in their paper [22].

The input data were the column vectors (time axis) of the space–time diagram, over which one-dimensional filtering was performed. Equation (1) describes such a filter:

(1) $$H\left(t\right)=\frac{1}{\sqrt{\pi }B}exp\left(-\frac{t^{2}}{B}\right)\cdot exp\left(j2\pi Ct\right),$$

where

- $exp^{j2\pi Ct}\equiv sin\left(2\pi Ct\right)$ is a harmonic function;
- *H(t)* is the filter function (kernel);
- *t* denotes time;
- *B* denotes throughput;
- *C* is the center frequency;
- $\frac{1}{\sqrt{\pi }B}$ is a normalizing factor.

In Figure 2, *ν* denotes the time frequency.

**Figure 3:**
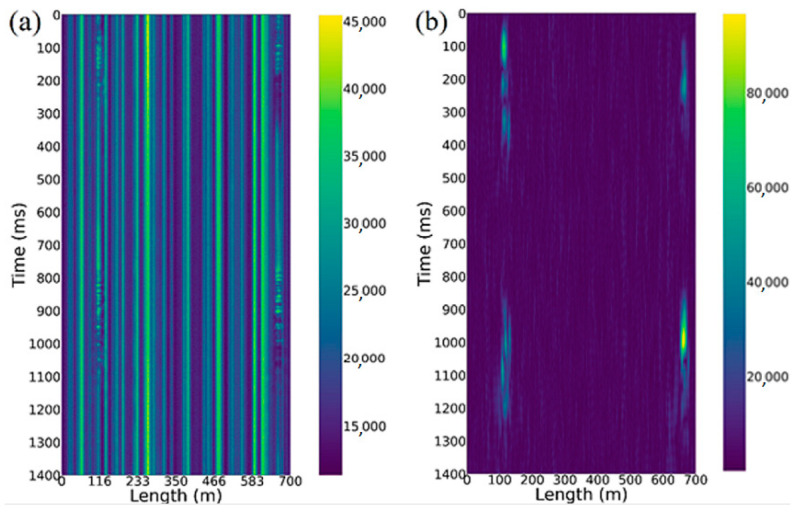
Visualization using a color map of (**a**) raw and (**b**) filtered charts.

Figure 3a shows an unfiltered space–time diagram, which records the signal associated with walking through snowdrifts along a distributed sensor at 110 and 660 m. In Figure 3b, the same pattern filtered with the designed band-pass filter is provided, which shows bright spots representing the acoustic footstep signals. Comparing the two figures, it can be seen that this filter can highlight such signals well.

**Figure 4:**
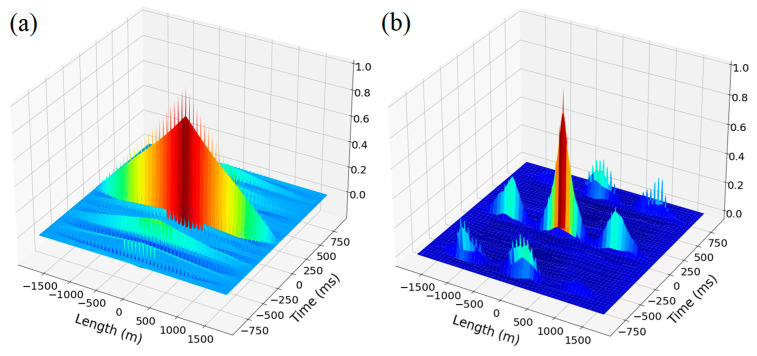
Visualization of a three-dimensional representation of the Pearson autocorrelation coefficient matrices: (**a**) raw and (**b**) filtered diagram.

To display the effectiveness of this filter, the Pearson autocorrelation coefficient matrices (Neto, A.M. et al. [23]) were calculated (see Figure 4), obtained using Equation (2).

(2) $$r_{ij}=\frac{\sum_{m}\sum_{n}\left[f\left(m+i,\;n+j\right)-\bar{f}\right]\left[f\left(m,\;n\right)-\bar{f}\right]}{\sum_{m}^{\;}\sum_{n}^{\;}{\left[f\left(m,\;n\right)-\bar{f}\right]}^{2}},$$

where

$r_{ij}$ denotes the Pearson autocorrelation matrix coefficient;

$\sigma_{f}$ is the standard deviation of *f*;

$\bar{f}$ is the mean of *f.*

From Figure 4, we can see that the signal-to-noise ratio (Stavrou, V.N. et al. [24]) of the filtered diagram is much higher than that of the raw diagram, which is beneficial in designing a classifier with high accuracy.

### 2.3. Pre-Processing

After filtering, the data enter the threshold condition block, in which 50 × 150 images are selected. The volume of an initial database can be increased (augmented), in order to improve the accuracy of predictions. Similar work has been carried out (Shi, Y. et al. [25]), conducting data augmentation using an adversarial network with cycle generation (CycleGAN). The essence of the algorithm used is that the existing data set is supplemented with modified existing data; for example, the images can be scaled, rotated at an angle, changed in terms of brightness distribution, and so on.

**Figure 5:**
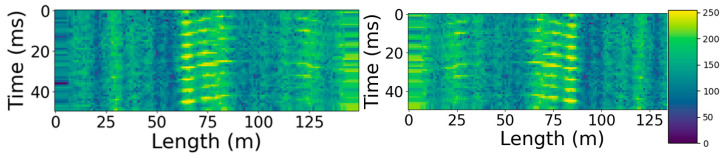
A set of symmetrically displayed matrices of acoustic signals of steps.

The most useful changes to the existing data set are vertical symmetric flipping (Figure 5) and changing the brightness of the images, as this does not violate the physical meaning of the signals. Afterwards, the received images are saved in png format, which does not lead to loss of information. For greater accuracy and a reliable assessment of the accuracy of predictions, the amount of data between classes is balanced. Thus, by generating new data, their volume is increased and the balancing of classes can be achieved.

### 2.4. Model Synthesis

#### 2.4.1. CNN-Based Architecture

A CNN is a hierarchical neural network consisting of a sequence of layers. A typical model generally consists of several convolutional layers, and the image content is represented as a set of feature maps obtained after convolution of the input data using various filters that are learned during the training phase. Selection layers (max-pooling) to obtain one value from several adjacent ones are introduced after convolutional layers, in order to reduce the image size and accumulate the maximum activation thresholds from the convolutional function maps. In addition, CNNs may also contain fully connected (FC) layers, where each neuron in the input layer is connected to each neuron in the adjacent layer. A sequence of convolutional layers, max-pooling layers, and FC layers forms a feature extraction pipeline that models the input data in an abstract form. Finally, a soft-max layer performs the final classification task, based on this representation.

**Figure 6:**
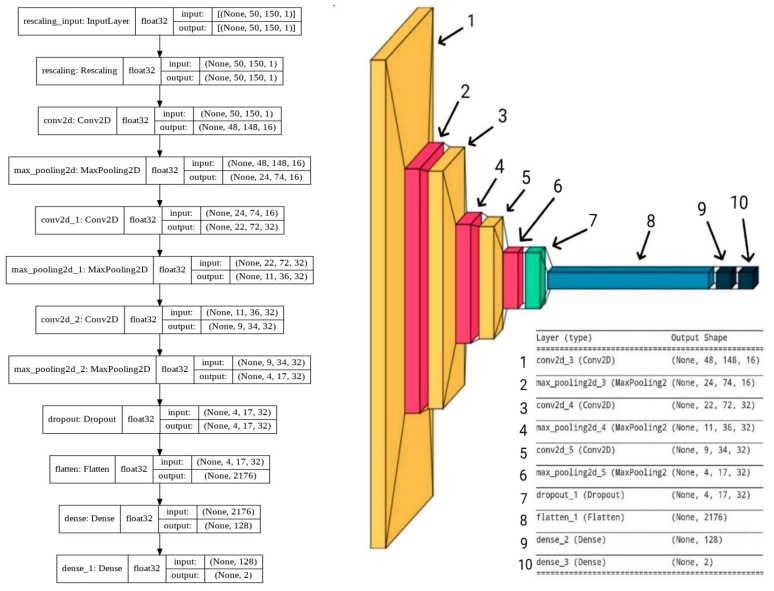
Architecture of the designed CNN.

When creating the CNN architecture, such factors, i.e., the low computational complexity of the system, were taken into account, affecting the simplification of the architecture and hyperparameters. The analyzed images of signals cannot be rotated, inverted, or significantly increased in scale, and have low complexity for generalization by the visual analyzer, which made it possible to simplify the CNN architecture. Images of signals have a low spatial hierarchy (Thakur, R. et al. [26]), for the generation of features of which three convolutional layers were considered sufficient. The basis for creating the architecture was AlexNet (Krizhevsky, A. et al. [27]). A smaller number of layers is not sufficient to allow for creation of the required number of templates (filters) to describe the depth of the spatial hierarchy of the classified images, while a larger number will not increase the accuracy of predictions but will increase the energy consumption. The created architecture (Figure 6) contains three convolutional layers, followed by the linear activation function ReLU described by Equation (3), which creates non-linearity in the intermediate calculations of the neural network.

(3) $$f\left(input\right)=Bool\;(input<0)\;\cdot input,\;$$

where

*Bool()* is the Boolean function;

*input* denotes the input data.

Max-pooling layers follow the linear activation functions of convolutional layers (Bieder, F. et al. [28]), which select the element with the highest value from four adjacent matrix elements. We propose the use of max-pooling as the signal images consist of light bands against a dark background, which makes it possible to highlight them. In addition, the higher the pixel values, the stronger their impact on the neural network. After passing the data through the max-pooling layer, a factor of 4 reduces their volume, which significantly reduces the computational costs of training without the loss of important information.

After the cascades of convolutional and max-pooling layers, the dropout layer follows, which excludes a certain percentage of random neurons. This operation is necessary to reduce the re-training of the neural network. Then, using the flatten layer, the tensor is stretched into one vector, which then passes to the first of two fully connected layers (FC), in which each output neuron is connected to all the input ones.

The first PS is followed by the second one, which has two exits. After each PS, there is a linear activation function. The soft-max activation function receives data from the second PS and returns the probabilistic class membership values, lying within the interval [0, 1]. This function is used as it gives greater accuracy compared to other functions.

#### 2.4.2. Neural Network Training

The learning optimizer is based on the stochastic gradient descent algorithm. The training data set is described by Equation (4):

(4) $$X=\left\{x_{i}|i=0,\ldots ,\;\left(N-1\right)\right\},$$

$$Y=\left\{y_{i}|i=0,\ldots ,\;\left(N-1\right)\right\},$$

where

*X* is the waveform image data set;

*Y* is the set of image labels;

$x_{i}$ denotes the *i*th image;

$y_{i}$ denotes the *i*th class label.

During the learning process, when the neural network makes a prediction, it is necessary to evaluate its accuracy. For this purpose, a loss function (Equation (5)) is introduced, which returns values proportional to the magnitude of the forecast inaccuracy:

(5) $$L\left(\omega \right)=\sum_{i=0}^{N-1}L\left(\omega ,\;x_{i},\;y_{i}\right),\;$$

where *ω* are the weights of the neural network.

It is necessary to select those weights at which the values of the loss function are as close as possible to zero. This problem is solved using the gradient descent method (Equation (6)). Some initial values for the weights are chosen. Then, we calculate the gradient of the loss function at this point ∇*L(ω_p_)* and shift the current point in the opposite direction of the gradient:

(6) $$\omega_{p+1}=\omega_{p}-\eta \nabla L\left(\omega_{p}\right),$$

where

∇ is the nabla operator;

$\omega_{p}$ denotes the weight values at the *p*th step. ∇

Repeating this process a sufficient number of times, we will reach the desired minimum of the function *L*(*ω_p_*). To control the speed of movement in the direction opposite to the gradient, an additional learning rate parameter, *η*, is introduced. For a sufficiently large data set, the calculation of the error function for all images in the set in one epoch is very expensive. Therefore, we divide the set into parts, with a batch size of 64.

The categorical cross-entropy was chosen as a loss function, the value of which depends on the degree of confidence for each class; that is, it is used to quantify the difference between two probability distributions (Equation (7)):

(7) $$H\left(P,\;Q\right)=-\sum_{x}P\left(x\right)\;log\left(Q\left(x\right)\right),\;$$

where

*P* is the distribution of true answers;

*Q* is the distribution of predictions.

## 3. Experimental Study and Discussion

**Figure 7:**
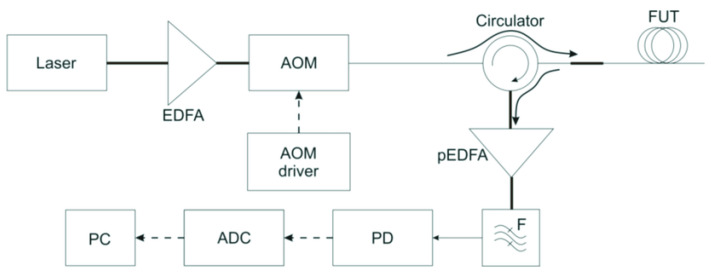
The experimental setup of a φ-OTDR system.

A phi-OTDR is a device that can detect acoustic impacts on a cable based on Rayleigh backscatter analysis (Stepanov, K. V. et al. [29]). The schematic of the device is shown in Figure 7. The radiation source is a frequency-stabilized narrow-linewidth laser, with a coherence length that is much greater than the pulse half-width ($\tau_{pulse}$~10…500 ns). This causes backscattered radiation interference in each pulse position. The continuous radiation from the laser is amplified by an erbium-doped fiber amplifier (EDFA). After the acousto-optic modulator (AOM) modulates the radiation to probing pulses, it passes to the sensor fiber through the circulator in the forward path. The backscattered radiation passes in the opposite direction in the circulator. Then, it is amplified by the pre-EDFA. Its amplified spontaneous emission (ASE) is excluded after it passes the narrow optical filter. Then, radiation enters the photodiode (PD) and is digitized on the analog-to-digital converter (ADC) before processing on the personal computer (PC).

**Figure 8:**
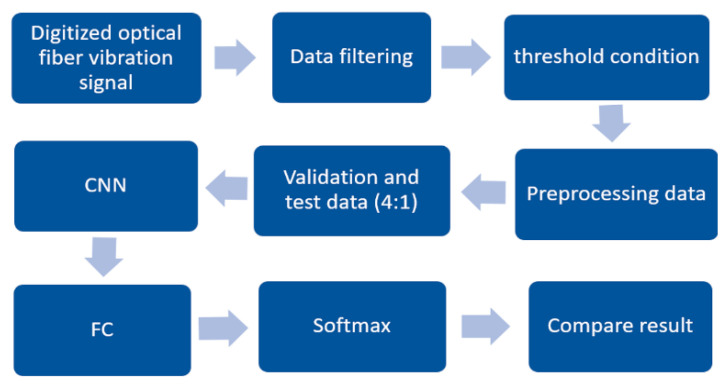
Block diagram of classifier training.

We conducted experiments regarding the registration of acoustic signals along a distributed sensor that is 40 km long, and data were obtained from the signals of steps and signals not related to steps. Step signals were extracted from the signals coming from the ADC using a wavelet filter. Having a priori information about events, after filtering, data were extracted for the data set, and the data were saved in two corresponding directories. False signals were obtained by repeatedly conducting experiments with the created classifier using one catalog with confirmed signals. Then, augmentation was carried out on the existing data set. For the experiment, 12,000 data were obtained. The training and validation data sets were split in a 4:1 ratio (Vrigazova, B., et al. [30]), respectively, and the amount of test data was 1000. The block diagram of model training is shown in Figure 8.

To select the optimal values for the hyperparameters of the created neural network architecture, the grid search method (GridSearchCV) was used (Ranjan GS, K. et al. [31]). GridSearchCV is an automatic fitting tool for machine learning models. GridSearchCV finds the best parameters by simple enumeration: a model is created for each possible combination of parameters. It is important to note that this approach can be very time-consuming. For the current work, a five-fold cross-validation grid search was carried out, according to the number of options for the optimization algorithm, the number of epochs, the number of filters in each convolutional layer, and the number of elements in the hidden fully connected layer (see Table 1).

An accuracy assessment algorithm was used to evaluate the resulting data, which calculated the score according to the number of correct answers when using the test data (500 samples of false signals and 500 confirmed ones). The algorithm returned the percentage of correct predictions.

Based on the learning curves of the trained neural network, the optimal value for the number of epochs was found, which was 60. For training, an ASUS GeForce GTX 1660 Ti graphics processor with 6 GB of RAM was used, which calculated a GPU-optimized algorithm (Tynchenko, V.S. et al. [32,33]).

**Figure 9:**
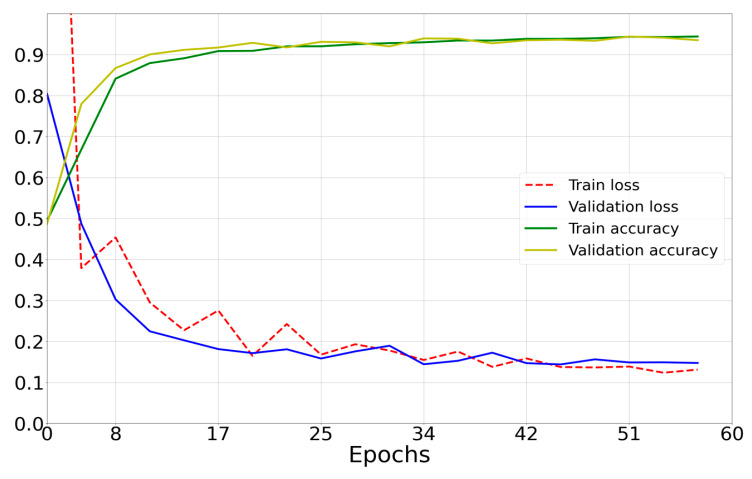
Loss curves of the created model (green and yellow) and accuracy curves (blue and red).

The main learning criterion in this work was the loss function (Figure 9).

The mean prediction calculation time was 0.032 s, the mean power consumption to perform one prediction was 400 mW, and the percentage of correct predictions was 96.91%.

**Figure 10:**
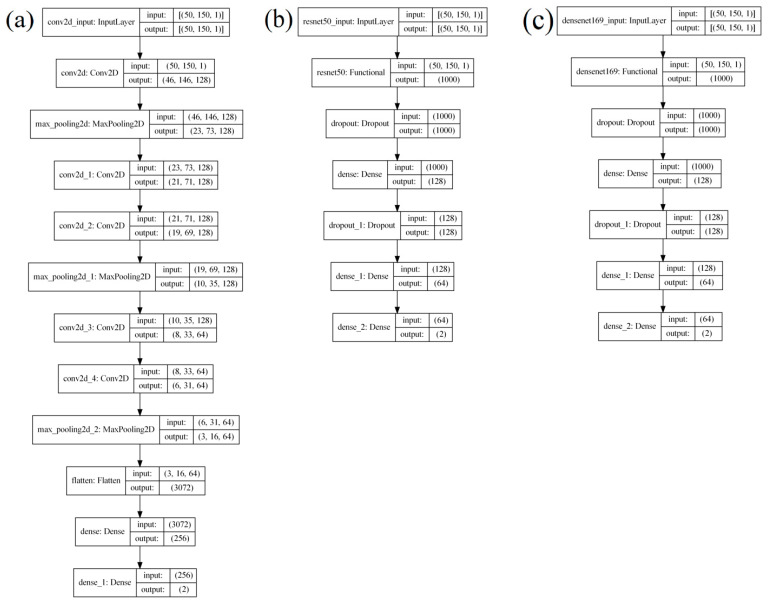
Compared architectures: (**a**) based on AlexNet; (**b**) based on ResNet-50; and (**c**) based on DenseNet-169.

We compared the percentage of correct predictions, the error matrix, the time taken to predict, and the registered power of the GPU during the execution of predictions on the created architecture under various popular architectures (Figure 10), such as ResNet-50, DenseNet-169, and the architecture based on AlexNet proposed in [4].

**Figure 11:**
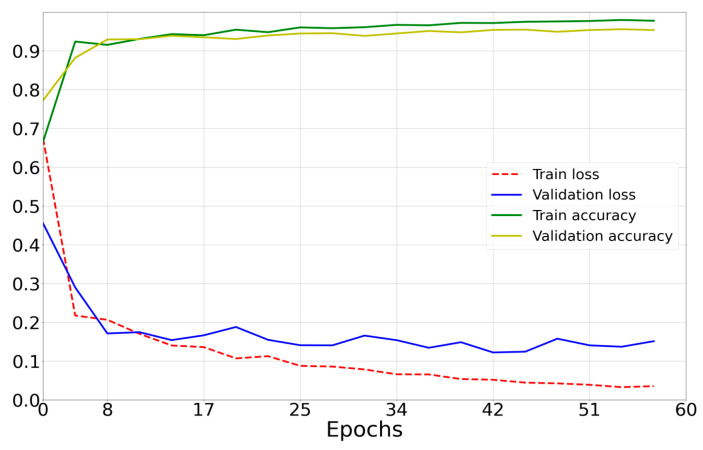
Loss curves of the model in Figure 9b (green and yellow) and accuracy curves (blue and red).

**Figure 12:**
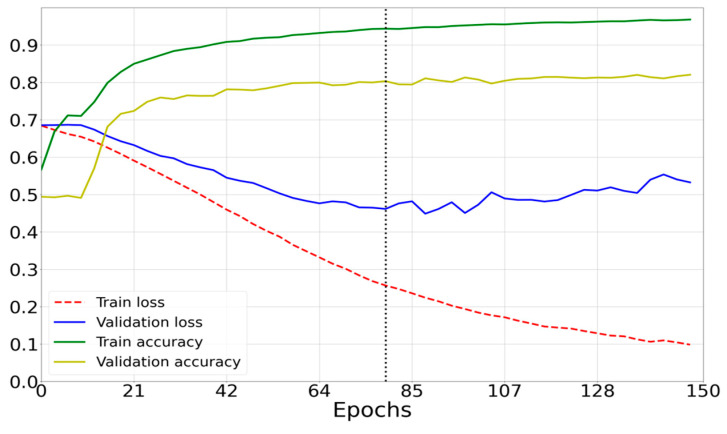
Loss curves of the model (Figure 9c) (green and yellow) and accuracy curves (blue and red).

**Figure 13:**
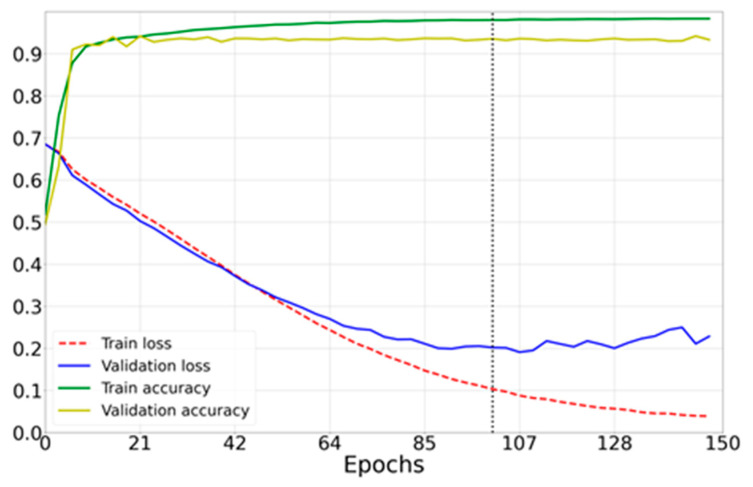
DenseNet169 model loss curves (green and yellow) and accuracy curves (blue and red).

Figure 11, Figure 12 and Figure 13 show the learning curves for the models compared in the work.

(1) Results for a neural network model with architecture based on AlexNet [4] (Figure 10a) are presented in Figure 11.
  - Number of epochs: 60;
  - Optimizer: Adam;
  - Mean prediction computation time: 0.037 s;
  - Mean power consumption to perform one prediction: 600 mW;
  - Percentage of correct predictions: 95.55%.
(2) ResNet50-based architecture results (Figure 10b) are presented in Figure 12.
  - Number of epochs: 90;
  - Optimizer: Adam;
  - Mean power consumption to perform one prediction: 3000 mW;
  - Mean prediction computation time: 0.086 s;
  - Percentage of correct predictions: 84.65%.
(3) Results of the architecture based on DenseNet169 (Figure 10c) are presented in Figure 13.
  - Number of epochs: 100;
  - Optimizer: Adam;
  - Mean power consumption to perform one prediction: 1700 mW;
  - Mean prediction computation time: 0.108 s;
  - Percentage of correct predictions: 88.85%.

**Figure 14:**
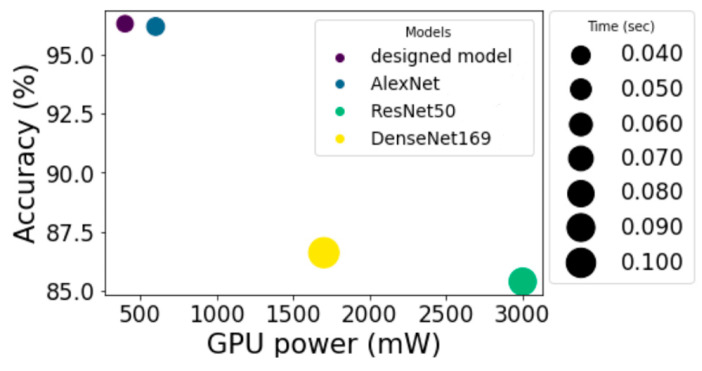
Comparison of the results of the studied and designed models.

Figure 14 compares the results of accuracy, power consumption, and time spent on making one prediction, and Table 2 compares the error matrices.

**Figure 15:**
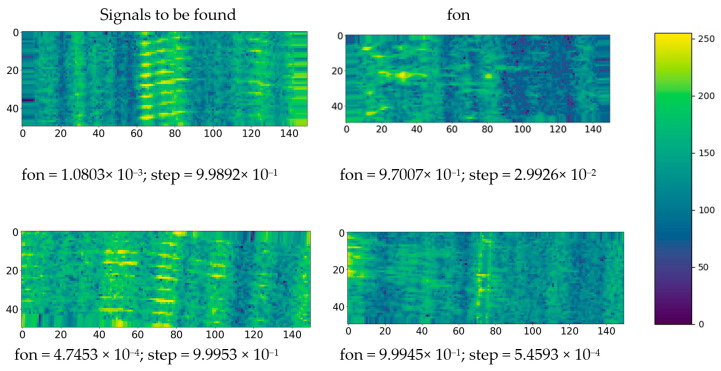
Example results of the created classifier on some data (grayscale images).

Figure 15 shows examples of two classes of some signals arriving as the input of the CNN and their probability of belonging to the classes.

Comparing the models, it can be seen that the use of more complex architectures increased the calculation time and energy consumption required to perform one prediction, as well as worsening the accuracy of predictions. This is due to the fact that complex CNN architectures are used to extract features from images with a sufficiently deep spatial hierarchy. In addition, with a more complex architecture, more data are required for training.

## 4. Conclusions

To extract useful information from the signals recorded by a phase-sensitive reflectometer, a wavelet filter was chosen and its optimal parameters were calculated, with which high-quality acoustic signal extraction was achieved.

A data set was created, consisting of two classes: the desired images of acoustic signals (steps) and acoustic images of various backgrounds. The most suitable ways to change the image parameters for data augmentation were studied and experimentally selected.

A classifier model based on a CNN architecture was created. The optimal hyperparameters of the model were selected through a five-fold cross-validation grid search considering a number of options: optimization algorithm, number of epochs, number of filters in each convolutional layer, and number of elements in the hidden fully connected layer. Based on the learning curves of the trained neural network, the optimal number of training epochs and learning rate were selected.

Thus, a classifier was obtained that could make predictions with sufficiently high accuracy while maintaining low computational complexity. In the course of comparing the characteristics obtained, through conducting experiments with neural network models based on the created and comparison architectures, we concluded that the created neural network model possesses the best characteristics.

In further studies, in order to increase the accuracy, reliability, and data invariance, the use of new algorithms for filtering and extracting the acoustic signals recorded by a phase-sensitive reflectometer will be considered.

## Figures and Tables

**Table 1:** Analyzed hyperparameters.

Optimizer	Adam	Adagrad	RMSprop
Number of filters in first layer	64	32	16
Number of filters in second layer	64	32	16
Number of filters in third layer	64	32	16
Number of neurons in the hidden fully connected layer	128	64	-

Grid search results: Optimizer, RMSprop. Number of filters in first layer, 64. Number of filters in second layer, 64. Number of filters in third layer, 64. Number of neurons in the hidden fully connected layer, 64.

**Table 2:** Confusion matrices of the studied models.

	Designed Model (Figure 6)		AlexNet (Figure 9a)	
	Actually Negative	Actually Positive	Actually Negative	Actually Positive
Predicted negative	98.46%	1.54%	91.92%	8.08%
Predicted positive	4.64%	95.36%	1.78%	98.22%
	ResNet50 (Figure 9b)		DenseNet169 (Figure 9c)	
	Actually Negative	Actually Positive	Actually Negative	Actually Positive
Predicted negative	82.7%	17.3%	95%	15%
Predicted positive	13.4%	86.6%	17.3%	82.7%

## Data Availability

Not applicable.
